# Incidence and Associated Factors of Laryngospasm among Pediatric Patients Who Underwent Surgery under General Anesthesia, in University of Gondar Compressive Specialized Hospital, Northwest Ethiopia, 2019: A Cross-Sectional Study

**DOI:** 10.1155/2020/3706106

**Published:** 2020-01-24

**Authors:** Wubie Birlie Chekol, Debas Yaregal Melesse

**Affiliations:** Department of Anesthesia, College of Medicine and Health Science, University of Gondar, Gondar, Ethiopia

## Abstract

**Introduction:**

Laryngospasm is a glottis closure due to reflex constriction of the laryngeal muscles. It can occur at any phase of the anesthetic. Different studies have been done previously with various results and indicative values which initiated us to do this research. This study aimed to assess the incidence and associated factors of laryngospasm among pediatric patients who underwent surgery under general anesthesia (GA).

**Methods:**

Institution-based, cross-sectional study was conducted on pediatric patients from February to August, 2019, in University of Gondar Comprehensive Specialized Hospital (UOGCSH). Data were entered and analyzed with SPSS version 20. Variables with *P* value less than <0.2 in bivariate analysis were fitted into the multivariable logistic regression analysis to identify factors associated with laryngospasm. Both crude and adjusted odds ratio with 95% CI were calculated to show strength of association. In multivariable analysis, *P* value of <0.05 was considered as statistically significant.

**Results:**

The incidence of laryngospasm among pediatric patients who underwent surgery under GA was 57 (18.4%). Of this, 34 (59.6%), 12 (21.1%), and 11 (19.3%) happened during emergence, maintenance, and induction phases of GA, respectively. In multivariable analysis, airway anomalies (AOR: 14.64, 95% CI: 1.71, 125.04), secretion (AOR: 2.45, 95% CI: 1.19, 5.06), attempts of airway devices (AOR: 2.47, 95% CI: 1.16, 5.22), upper respiratory tract infection (AOR: 2.91, 95% CI: 1.008, 8.41), and inadequate depth of anesthesia (AOR: 7.92, 95% CI: 2.7, 23.22) were significantly associated with incidence of laryngospasm.

**Conclusions:**

Laryngospasm can occur at any phase of the anesthetic. At UOGCSH, the overall rate of laryngospasm was 18.4%, with the vast majority of episodes occurring on emergence. Inadequate depth of anesthesia, URTI, airway anomalies, multiple attempts of airway devices, and oropharyngeal secretion were predictors of laryngospasm. So, added vigilance is needed in patients with URTI, airway anomalies, or those who require multiple attempts at airway device insertion. Prompt clearing of airway secretions and adequate depth of anesthesia may help to prevent laryngospasm. Since the majority of our patients received an IV induction, endotracheal intubation, and maintenance with halothane, caution must be taken in extrapolating these results to other patient populations.

## 1. Introduction

Pediatric laryngospasm is defined as glottis closure due to reflex constriction of the laryngeal muscles that produce partial or complete obstruction of the larynx. When complete and sustained, laryngospasm can become an anesthetic emergency. It mainly happens at induction, maintenance, and emergence phases of GA [1]. Laryngospasm usually manifests with the sign of inspiratory stridor which may progress to complete obstruction, increased respiratory effort, tracheal tug, paradoxical respiratory effort, oxygen desaturation with or without bradycardia, and airway obstruction which does not respond to a Guedel airway [2].

The reported incidence of laryngospasm was differing in different studies. Laryngospasm more commonly happens in pediatric anesthetic practices than adults. The overall incidence of laryngospasm was 0.87% in adults, 1.7% in pediatrics, and 2.82% in infants. The incidence of laryngospasm in older children was twice that of adults, while the incidence of laryngospasm in younger children was three times that of adults [3–5]. According to Haile et al.'s report, the incidence of laryngospasm on pediatric patients who underwent surgery under GA was 28.3% [6]. Another study reported the incidence of laryngospasm as 10% in the very young pediatric patients with reactive airways, either due to upper respiratory tract infection or asthma. The incidence of laryngospasm has been reported in the literature as high as 25% in patients undergoing tonsillectomy and adenoidectomy [7].

The main triggering factors for laryngospasm were inappropriate depth of anesthesia, suction catheter, thiopentone, inhalational induced irritation, secretion, stimulation of the airway, tracheal intubation [8], airway anomalies, and upper respiratory tract infection [9, 10]; surgical factors include tonsillectomy, adenoidectomy, appendiectomy, dilatation of the anus, and thyroidectomy [11–13].

If laryngospasm persists, it may cause to hypoxia and hypercapnea. In rare situations, serious morbidity and mortality of hypoxia and hypercapnea may lead to arrhythmia, aspiration of gastric content, bronchospasm, and cardiac arrest [1, 14].

According to Haile et al.'s study, laryngospasm was managed by stimulus removal, continuous positive airway pressure (CPAP) with 100% oxygen, jaw thrust, and increasing the depth of anesthesia for partial laryngospasm and administration of intravenous suxamethonium for complete laryngospasm [6].

The quality of anesthesia gets improved through time but still we had personal observations about the incidence of laryngospasm in pediatrics population. Also, several adverse respiratory features were encountered in concomitant with laryngospasm. Although considerable research studies have been done previously on the consequences of laryngospasm, varied results on previous studies and indicative values of previous studies initiated us to do this research. So, this study was done to determine the incidence and associated factors of laryngospasm in pediatric patients who underwent surgery under GA at UOGCSH.

## 2. Methods and Materials

### 2.1. Study Design, Period, and Study Settings

Institutional-based cross-sectional study was conducted on pediatric patients that underwent surgery under GA in UOGCSH from February 1 to August 1, 2019. This hospital is located in Amhara region, North Gondar Zone, which is about 738 km away from Addis Ababa in Northwest of Ethiopia.

### 2.2. Study Population

All consecutive pediatric patients (birth–12 years) that were operated upon emergency and elective conditions with GA at the main and ophthalmic operation theatres during the study period were included.

### 2.3. Variables of the Study

The outcome variable was incidence of laryngospasm. The independent variables were sociodemographic factors (age, sex, and ASA status), patient factors (current history of URTI, comorbidities, preexisting airway anomalies, and history of exposure to smoke inhalation), anesthetic factors (types of anesthesia, use of oral airway devices, types of induction, induction drugs, maintenance drugs, multiple attempts of airway device insertion, inadequate depth of anesthesia, and secretion at oropharynx), and surgical factors (types of surgery and urgency of surgery).

### 2.4. Definitions of Terms Used in This Study

Laryngospasm: glottic closure due to reflex constriction of the laryngeal muscles that produce partial or complete obstruction of the larynx that manifested either alone or in combination of inspiratory stridor, increased respiratory effort, tracheal tug, paradoxical respiratory effort, desaturation, and bradycardia [2].

Signs of inadequate depth of anesthesia: when patients were manifest any of the following movements, increased respiratory rate, increased heart rate, or increased blood pressure in response to stress or painful stimuli [15].

Multiattempts of airway device: defined as ≥2 attempts in securing of the airway [16].

### 2.5. Sample Size Determination

The sample size was determined by using a single proportion formula (*N*=(( *z*/2 )^2^*xpq*)/*d*^2^) with a study done in Jimma University Specialized and Teaching Hospital (2015), Southwest Ethiopia, which reported that the incidence of laryngospasm was 28.3% [6] by assuming 95% confidence level, 5% margin of error, and 5% none response rate, where *N* = sample size, *P* = percentage, *Q* = 1 − *P*, *D* = desired degree of precision, and *Z* = the standard normal value at the level of confidence desired. *N* = (1.96)^2^ (0.72) (0.28)/(0.05)^2^ = 309. When 5% of the nonresponse rate was added, the total number of patients who participated in the study was *N* = 325.

### 2.6. Data Management and Collection Procedures

Training was given for data collectors and supervisors. The training was focused on each item of the study tool, relevance of the study, and how to ensure confidentiality of patient's data. The data collection procedures were including consent from the parents, reviewing of the chart, interviewing of the parents, recording of anesthetic factors, surgical factors, interventions done for cases, and laryngospasm incidence at induction, maintenance, and emergence phases of GA. During data collection, regular supervision and follow-up were done for the completeness, accuracy, and clarity of data.

### 2.7. Data Analysis and Interpretation

SPSS version 20 was used for data entry and analysis. Both bivariate and multivariate binary logistic regression analyses were used to identify factors associated with laryngospasm. Variables with *P* value less than <0.2 in the bivariate analysis were fitted into the multivariable logistic regression analysis. Both crude and adjusted odds ratio with the corresponding 95% CI were calculated to show the strength of association. Hosmer and Lemeshow test was used to assess the goodness of fit. In multivariable analysis, variables with *P* value of <0.05 were considered as statistically significant. Categorical variables were analyzed with chi-square test and presented with frequency (percent). Tables and graphs were used for presentation of descriptive data.

### 2.8. Ethical Consideration

Ethical clearance was obtained from the Ethical Review Board of School of Medicine at University of Gondar. Informed consent was obtained from the parents. Brief explanation for parents was done about the risks and benefits in study participation. Confidentiality and anonymity were ensured.

## 3. Results

### 3.1. Sociodemographic and Patient-Related Variables

A total of 310 patients were included in this study with 95.4% response rate. Fifteen patients were excluded from analysis due to the incomplete data. The study involved 177 (55.2%) male and 139 (44.8) female participants with age range from birth to 12 years. Majority of study participants 238 (76.8%) were ASA I, while the remaining 56 (18.1%) and 16 (5.1%) were ASA II and ASA III, respectively. In this study, 9 (2.9%) of the study participants were having airway anomalies, 13 (4.2%) of patients were having respiratory comorbidities, and 33 (10.64%) of patients were having URTI.

### 3.2. Surgical- and Anesthetic-Related Variables

Majority of patients 195 (62.9%) were operated under GA with ETT. The commonly done procedures in this study were abdominal 103 (33.2%), ophthalmic 67 (21.6%), orthopedic 50 (16.1%), and ENT 44 (14.2%) procedures (Table 1).

### 3.3. Anesthetic-Related Variables

Majority of the study participants 270 (87.1%) were induced with intravenous anesthetic agents. On the other hand, greater part of them 210 (67.7%) were maintained with halothane (Table 2).

### 3.4. Factors Associated with the Incidence of Laryngospasm

In this study, pediatric patients who operated under GA with having URTI were about 2.91 times (AOR: 2.91, 95% CI: 1.008, 8.41), more likely to have laryngospasm when compared with no URTI. Having of airway anomalies at preoperative assessment was 14.6 times (AOR: 14.64, 95% CI: 1.71, 125.04), more likely to have laryngospasm than those who were not having airway anomalies. Similarly, those patients who had the signs of inadequate depth of anesthesia were 7.92 times (AOR: 7.92, 95% CI: 2.70, 23.22) more likely to develop laryngospasm than no manifestations (Table 3).

### 3.5. Incidence of Laryngospasm

The overall incidence of laryngospasm was reported as 57 (18.4%). Of the total incidence of laryngospasm, 34 (59.6%), 12 (21.1%), and 11 (19.3%) happened during emergence, maintenance, and induction phases of GA, respectively.

### 3.6. Complications after Laryngospasm

Among the 57 (18.4%) laryngospasm events, desaturation occurred in 56 (98.3%), bradycardia occurred 54 (94.8%), decreased air entry occurred in 46 (80.7%), increased inspiratory effort occurred in 20 (35.1%), paradoxical breathing occurred in 22 (38.6%), and cyanosis occurred in 7 (12.3%) of laryngospasm cases.

### 3.7. Management for Laryngospasm

Among the 57 (18.4%) laryngospasm events, the spasm was broken with removing the offending stimulus and administration of CPAP with 100% oxygen for 37 (64.9%) of events, increased the depth of anesthesia for 7 (12.3%) events, and administration of suxamethonium for 5 (8.8%) of laryngospasm events.

## 4. Discussion

This study was conducted to find out the incidence and associated factors of laryngospasm at induction, maintenance, and emergence phases of GA. The overall incidence of laryngospasm was 57 (18.4%). The incidence of this study was slightly lower than a study done by Haile et al. (28.3%); however, it was still higher than studies done by Orestes et al. (1.6%), Peng et al. (12.5% with LMA, 9.5% with ETT), and Al-Metwalli et al. (8%) [17–19]. The discrepancies could be due to the modern refinements in anesthesia, surgical techniques, and specificity of the procedures in the previous studies.

Of the overall incidence, 34 (59.6%), 12 (21.1%), and 11 (19.3%) happened during emergence, maintenance, and induction phases of GA, respectively. Haile et al. found out that the overall incidence of laryngospasm was 53 (28.3%), of which 30 (56.6%) happened during induction, 4 (7.6%) during maintenance, and 19 (35.8%) happened during emergence phases of GA [6]. The possible explanation for the high incidence of laryngospasm during emergence in our study might be due to the high percentage of patients operated under GA with ETT 195 (62.9%). This suggestion was supported by a study done by Visvanathan et al. who found that the incidence of laryngospasm occurred more on the induction phase if the patient was on LMA and high during emergence if the patient was on ETT [20].

Regarding to the associated factors, having URTI in pediatric surgical patients who operated under GA was about 2.91 times (AOR: 2.91, 95% CI: 1.008, 8.41), more likely to had laryngospasm when compared with no URTI. This study was supported by studies done in different settings which reported that there was statistically significant association between URTI and laryngospasm among pediatric patients who operated under GA [5, 10, 21–25]. However, prospective studies have been failed to conclusively demonstrate an association between laryngospasm and URTI [26].

Having of airway anomalies at preoperative assessment was 14.6 times (AOR: 14.64, 95% CI: 1.71, 125.04), more likely to had laryngospasm than those who were not having airway anomalies. This finding was in line with other studies done by Flick et al. and Cohen et al. [10, 23].

This study verified that intravenous anesthetic induction was used as protective agents for laryngospasm when compared with those induced with inhalational anesthetic agents (AOR: 0.31, 95% CI: 0.12, 0.78). In agreement with this study, different literatures reported that propofol had less risky of laryngospasm due to the ability of blunting the airway reflexes, and ketamine had no laryngospasm effect unless the increased tendency of secretion. However, inhalational anesthetic agents including desflurane and isoflurane were having higher incidences of laryngospasm in pediatric age groups [8, 27, 28].

Having the signs of inadequate depth of anesthesia was 7.92 times (AOR: 7.92, 95% CI: 2.70, 23.22), more likely to develop laryngospasm than no manifestations. In congruent with this, a study done by Aaalami et al. confirmed that inadequate depth of anesthesia had a significant effect on the incidence of laryngospasm [5, 29].

Those pediatric patients who had secretion at the oropharynx were 2.45 times (AOR: 2.45, 95% CI: 1.19, 5.06), more likely to develop laryngospasm than who had no secretion at the oral cavity. This finding was supported by Olsson et al's. study [5].

Attempting of multiple times during airway devices insertion was 2.47 times (AOR: 2.47, 95% CI: 1.16, 5.22), more likely to develop laryngospasm than no multiple attempts. In matching with this finding, Flick et al. and Hernandez et al. identified the association between multiple attempts of airway devices insertion and laryngospasm [10, 30].

Finally, the study was having some limitations including the diagnosis of laryngospasm was only dependent on the clinical signs and there was no specification whether the laryngospasm is partial or complete.

## 5. Conclusion

Laryngospasm can occur at any phase of the anesthetic. At UOGCSH, the overall rate of laryngospasm was 18.4%, with the vast majority of episodes occurring on emergence. Inadequate depth of anesthesia, URTI, airway anomalies, multiple attempts of airway devices and oropharyngeal secretion were predictors of laryngospasm. So, added vigilance is needed in patients with URTI, airway anomalies, or those who require multiple attempts at airway device insertion. Prompt clearing of airway secretions and adequate depth of anesthesia may help to prevent laryngospasm. Since the majority of our patients received an IV induction, endotracheal intubation, and maintenance with halothane, caution must be taken in extrapolating these results to other patient populations.

## Figures and Tables

**Table 1:** Surgical- and anesthetic-related variables of pediatric patients who underwent surgery under GA in UOGCSH, February 1–August 1, 2019 (*N* = 310).

Variables	Frequency (*n*)	Percent (%)	Laryngospasm	
			Yes (*n*)	No (*n*)
Procedures				
Ophthalmic	67	21.6	13	54
Anal	7	2.3	4	3
Abdominal	103	33.2	16	87
Neurosurgery	20	6.5	2	18
ENT	44	14.2	13	31
Orthopedic	50	16.1	5	45
Foreign body	19	6.1	4	15
Types of anesthesia				
GA with ETT	195	62.9	40	155
GA with LMA	31	10.0	6	25
GA with facemask	84	27.1	11	73
Urgency of surgery				
Emergency	163	52.6	25	138
Elective	147	47.4	32	115
Induction agents				
Ketamine	116	37.4	16	100
Thiopental	26	8.4	10	16
Propofol	47	15.2	5	42
Ketofol	81	26.1	13	68
Halothane	36	11.6	11	25
Isoflurane	4	1.3	2	2

**Table 2:** Anesthetic-related variables of pediatric patients who underwent surgery under GA in UOGCSH, February 1–August 1, 2019 (*N* = 310).

Variables	Frequency (*n*)	Percent (%)	Laryngospasm	
			Yes (*n*)	No (*n*)
Maintenance agents				
Halothane	210	67.7	40	170
Isoflurane	84	27.1	13	71
Ketamine	8	2.6	1	7
Propofol	3	1.0	2	1
Thiopental	2	0.6	0	2
Ketofol	3	1.0	1	2
Multiattempt airway				
Yes	134	43.2	40	94
No	176	56.8	17	159
Inadequate depth anesthesia				
Yes	30	9.7	20	10
No	280	90.3	37	243
Types of induction				
Intravenous	270	87.1	44	226
Inhalational	40	12.9	13	27
Oropharyngeal secretion				
Yes	96	31.0	34	62
No	214	69.0	23	191
Oral airway used				
Yes	14	4.5	7	7
No	296	95.5	50	246

**Table 3:** Factors fitted into the multivariable logistic regression analysis in pediatric patients who underwent surgery under GA in UOGCSH, February 1–August 1, 2019 (*N* = 310).

Variables	Laryngospasm		Odds ratio	
	Yes, *n* (%)	No, *n* (%)	Crude (95% CI)	Adjusted (95% CI)
Urgency of surgery				
Emergency	25 (15.3)	138 (84.7)	0.65 (0.36, 1.16)	0.62 (0.29, 1.30)
Elective	32 (21.8)	115 (78.2)	1	1
Airway anomalies				
Yes	7 (77.8)	2 (22.2)	17.57 (3.55, 87.07)	14.64 (1.71, 125.04)^*∗*^
No	50 (16.6)	251 (83.4)	1	1
URTI				
Yes	21 (63.6)	12 (36.4)	11.72 (5.31, 25.84)	2.91 (1.008, 8.41)^*∗*^
No	36 (13)	241 (87)	1	1
Oral airway				
Yes	7 (50)	7 (50)	4.92 (1.65, 14.65)	1.47 (0.36, 6.03)
No	50 (16.9)	246 (83.1)	1	1
Types of induction				
IV	44 (16.3)	226 (83.7)	0.40 (0.19, 0.84)	0.31 (0.12, 0.78)^*∗*^
Inhalational	13 (32.5)	27 (67.5)	1	1
Inadequate depth anesthesia				
Yes	20 (66.7)	10 (33.3)	13.13 (5.70, 30.25)	7.92 (2.70, 23.22)^*∗*^
No	37 (13.2)	243 (86.8)	1	1
Oropharyngeal secretion				
Yes	34 (35.4)	62 (64.6)	4.55 (2.49, 8.31)	2.45 (1.19, 5.06)^*∗*^
No	23 (10.7)	191 (89.3)	1	1
Comorbidities				
Yes	6 (46.2)	7 (53.8)	4.13 (1.33, 12.82)	1.99 (0.35, 11.24)
No	51 (17.2)	246 (82.8)	1	1
Multiattempt airway				
Yes	40 (29.9)	94 (70.1)	3.98 (2.14, 7.41)	2.47 (1.16, 5.22)^*∗*^
No	17 (9.7)	159 (90.3)	1	1

*Note.* ^*∗*^ *P*-value <0.05 was considered as statistically significant.

## Data Availability

The data sets used and analyzed during the study are available from the corresponding author on reasonable request.

## Abbreviations

ASA: American Society of Anesthesiologists
CPAP: Continuous positive airway pressure
ENT: Ear, nose, and throat
ETT: Endotracheal tube
GA: General anesthesia
LMA: Laryngeal mask airway
UOGCSH: University of Gondar Comprehensive Specialized Hospital
URTI: Upper respiratory tract infection.
